# Effects of Micro-Topography and Vegetation on Soil Moisture on Fixed Sand Dunes in Tengger Desert, China

**DOI:** 10.3390/plants13111571

**Published:** 2024-06-06

**Authors:** Dinghai Zhang, Youyi Zhao, Haidi Qi, Lishan Shan, Guopeng Chen, Ting Ning

**Affiliations:** 1 Centre for Quantitative Biology, College of Science, Gansu Agricultural University, Lanzhou 730070, China; zhaoyy@gsau.edu.cn (Y.Z.); qhd17539@163.com (H.Q.); 18089489751@163.com (T.N.); 2 College of Forestry, Gansu Agricultural University, Lanzhou 730070, China; shanls@gsau.edu.cn (L.S.); chgp1986@gmail.com (G.C.)

**Keywords:** sand-binding vegetation, soil moisture, micro-geomorphic units, topographic factors, vegetation factors, Tengger Desert

## Abstract

Soil moisture is a key factor in arid ecosystems, with local variations influenced by topography and vegetation. Understanding this relationship is crucial for combating desertification. Employing ANOVA, Mean Decrease Accuracy (MDA) analysis from random forest modeling and Structural Equation Modeling (SEM), this study investigates the distribution of soil moisture and its associations with topographic and vegetative factors across four micro-geomorphic units in the Tengger Desert, China. Significant heterogeneity in soil moisture across various layers and locations, including windward and leeward slopes and the tops and bottoms of dunes, was observed. Soil moisture generally increases from the surface down to 300 cm, with diminishing fluctuations at greater depths. Soil moisture peaks in the surface and middle layers on windward slopes and in deep layers at the bottom of dunes, exhibiting an initial rise and then a decline on windward slopes. Topographic (including slope direction and elevation difference) and vegetation (including shrub and herb coverage) factors significantly influence soil moisture across three depth layers. Topographic factors negatively affect soil moisture directly, whereas vegetation positively influences it indirectly, with shrub and herb abundance enhancing moisture levels. These insights inform ecological management and the formulation of soil moisture-conservation strategies in arid deserts. The study underscores customizing sand-binding vegetation to various micro-geomorphic dune units.

## 1. Introduction

Sandstorm hazard areas in China are primarily located between longitudes 75° and 125° and latitudes 35° and 50°, encompassing extreme arid, arid, semi-arid and semi-humid regions across various bioclimatic zones. The most severe desertification in China occurs in the sand lands and the farming-pastoral ecotones of eastern China, where average annual precipitation exceeds 250 mm, and in the transition zones between desert oases and desert grasslands west of Helan Mountain, where it is less than 200 mm [1]. Soil moisture is a crucial ecohydrological variable that affects the stability and functioning of arid and semi-arid ecosystems [2,3,4]. The status and distribution of soil moisture impact hydrological processes such as infiltration, runoff and evaporation, and also influence plant establishment, growth, mortality, morphology, transpiration and photosynthetic rates on an individual scale. On a community scale, soil moisture affects vegetation type, distribution, structure and composition, while on a regional scale, it influences vegetation patterns, landscape distribution, competitive interactions and succession processes [5,6]. However, the factors influencing soil moisture are complex and highly nonlinear, impacted by biotic and abiotic variables including climate, topography and vegetation. Additionally, the types and structure of vegetation and community composition contribute to spatiotemporal variation in soil moisture. The coupling and mutual feedback between soil moisture and plants further complicate the study of their interaction.

Plants play a crucial role in the movement of soil moisture back to the atmosphere. Rainfall captured by plant canopies may either evaporate or convert into throughfall and stemflow, thereby replenishing soil moisture [7]. The percentage of gross rainfall that becomes throughfall and stemflow typically ranges from 70 to 90% [8,9,10]. Plants form preferential flow pathways via both active and recently deceased roots, which concentrate water at tree bases through stemflow and enhance soil infiltration [11,12,13]. Additionally, they extract soil moisture from inter-canopy areas [14,15], which leads to competition for water among plants and influences the formation of vegetation patterns [16]. Soil moisture dynamics in the root zone have critical effects on various plant processes, such as nutrient transport, photosynthesis and hormone transport, which are essential for plant growth and community composition [17,18,19]. It is crucial to understand the future dynamics of soil moisture and their ecohydrological impacts on adapting to climate change. While soil moisture modeling approaches can assist in predicting future trends, a thorough understanding of soil moisture–plant interactions and associated ecohydrological processes is essential for the effective utilization of these models.

Topography has long been recognized as a significant factor influencing vegetation patterns at local scales, primarily due to the close relationship between elevation and temperature [20,21,22]. Since the 1890s, it has been known that micro-topography impacts local vegetation patterns through its influence on the surrounding abiotic environment [23]. Micro-topography plays a crucial role in determining local plant distribution and diversity patterns across various habitats, including forests, marshes and mangroves, tropical rainforests, grasslands, deserts and savannahs [24,25,26,27]. Furthermore, robust evidence indicates that distinct plant communities are found at different micro-topographic sites in the Great Hungarian Plain and in permanent dune grasslands in northwestern Wales [26,28]. All of the authors hypothesized that these results were caused by topographically controlled soil moisture. A recent study on health and dry grassland plants on rock outcrops in eastern Germany revealed a strong correlation between the floristic gradient and both soil moisture and nutrient availability, suggesting that micro-topography regulates these elements [29]. Micro-topography plays a critical role in determining local patterns in incident solar energy [30]. In arid ecosystems, variations in soil moisture, mainly influenced by topographically controlled solar radiation, also affect local vegetation patterns. However, the mechanisms and extent of micro-topography’s influence on soil moisture at smaller spatial scales—where elevation changes are insufficient to induce temperature variations—remain poorly understood.

Understanding the interactions between soil moisture, vegetation and topography is crucial for ecohydrology research in desert ecosystems, particularly at the local scale. By investigating these relationships, including the extent and pathways of influence, soil moisture models with detailed process representation and feedback mechanisms can be improved. However, the effects of vegetation and topography on soil moisture at the local scale have not been thoroughly assessed, particularly in desert ecosystems. Further research is needed on the distribution characteristics of soil moisture across various micro-geomorphic units within sand dunes, as well as on evaluating both the direct and indirect effects of topographic and vegetation factors on soil moisture levels. This study assumes that micro-geomorphic factors exert direct and indirect influences on soil moisture. These factors may directly alter soil moisture levels or indirectly modulate them through shrub and herbaceous components. Furthermore, it is postulated that interactions occur between shrub and herbaceous factors, both capable of impacting soil moisture dynamics. Our study aims to examine the distribution characteristics and variations of soil moisture across three depth layers and four micro-geomorphic unit types in fixed sand dunes of the Tengger Desert in China. Furthermore, we seek to identify the primary topographic and vegetation factors influencing soil moisture and to assess the magnitude and direction of their impacts on soil moisture levels. Addressing these important questions is essential for successfully executing extensive revegetation initiatives in arid and semi-arid desert ecosystems, which are crucial in combating desertification.

## 2. Materials and Methods

### 2.1. Site Description 

**Figure 1:**
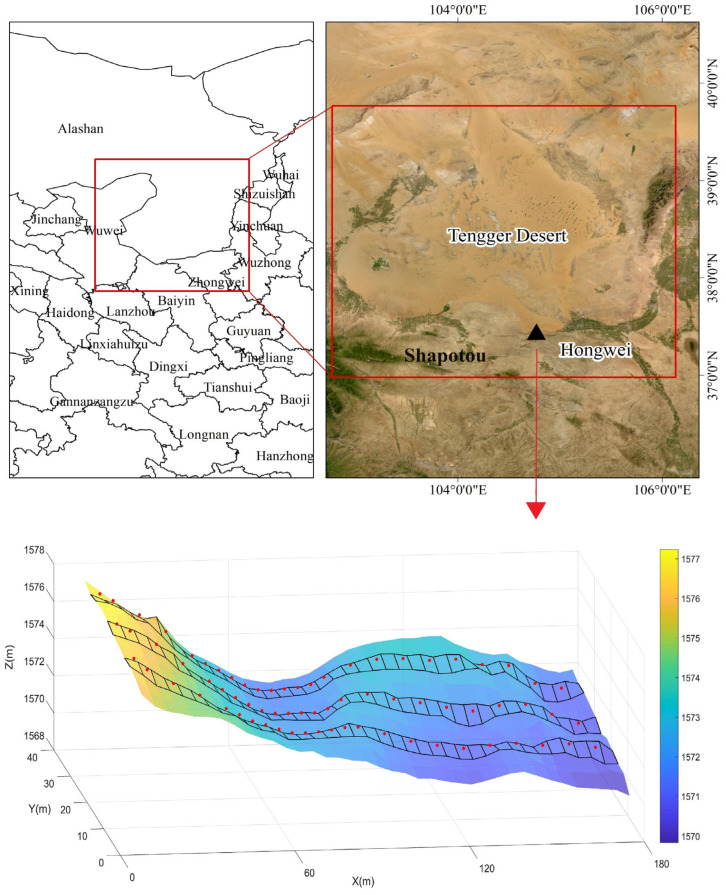
The location of the study area and experimental plot on a fixed sand dune. The checkered sampling belt is divided into three zones for soil moisture sampling; a red dot marks the specific location of a soil moisture sampling point. Variations in color shades across the plot indicate elevation changes.

The study area is located in the Hongwei region, which is situated at the southeastern margin of the Tengger Desert in China (Figure 1). It spans between north latitudes 37°30′~40°10′ N and east longitudes 102°20′~105°55′ E, with an altitude range from 1200 to 1400 m, covering approximately 5922.4 km^2^. The region experiences an average annual temperature of approximately 10.0 °C, ranging from a January low of −6.1 °C to a July high of 24.7 °C. The average annual rainfall is 176.5 mm, with about 80% occurring between June and September [31]. Rainfall events typically feature small quantities and low intensities, with nearly 64% of events registering less than 0.5 mm/h. Groundwater depth ranges from 50 to 80 m, making precipitation the sole reliable water source for sand-binding vegetation. The average annual wind speed is 2.9 m/s, and the region receives 3264 h of sunshine per year. There are approximately 59 sandstorm days per year, with annual evaporation rates ranging from 1500 to 2000 mm. For the soil in the study area, clay particles are sparsely distributed within the 0–15 cm depth range, with sand particles being the predominant component, followed by silt particles. The proportion of very fine sand (50–100 μm) and fine sand (100–250 μm) is notably high, ranging from 81.50% to 87.79% and from 9.37% to 17.96%, respectively, with mean values of 85.79% and 12.25% [32]. Conversely, the silt particle content (2–50 μm) is comparatively low, averaging 2.50%. For soil below 15 cm depth, sand particles overwhelmingly dominate the soil composition, with very fine sand and fine sand collectively exceeding 97%. Soil moisture content varies between 0.4% and 3.0% by weight [31]. The region features extensive lattice and barchan dune chains, with sand-binding vegetation predominantly found on fixed and semi-fixed sand dunes. Vegetation coverage on fixed sand dunes can reach 15 to 55%, densely vegetated with shrubs, semi-shrubs and herbaceous plants. The main sand-binding shrubs on fixed sand dunes include *Artemisia ordosica*, *Caragana korshinskii*, *Ceratoides latens*, *Oxytropis aciphylla*, *Caragana microphylla*, *Calligonum arborescens* and *Atraphaxis bracteate* [1].

### 2.2. Field Investigation and Experimental Design

In July 2023, an experimental plot was established on fixed sand dunes in the Hongwei area of the Tengger Desert (104°46′01″ E, 37°27′00″ N), consisting of four types of micro-geomorphic units: leeward slope, windward slope, dune top and dune bottom. The experimental plot, measuring 40 × 180 m, was subdivided into 450 smaller quadrats of 4 × 4 m each, arranged in a 10 × 45 grid (Figure 1). Soil moisture sampling focused on rows 3, 6 and 9 to target areas with significant topographic relief on the sand dunes. A total of 72 soil moisture sampling points were distributed across the experimental plot. Measurements were conducted at varying depths from 0 to 300 cm, segmented into 18 layers (0–5 cm, 5–15 cm, 15–25 cm, 25–35 cm, 35–50 cm, followed by 20 cm intervals beyond 50 cm). Soil moisture samples were collected using custom-made sand augers and transferred into sealed soil containers. These containers were then transported to the laboratory for weighing on a high-precision electronic balance fitted with a glass cover. Subsequently, the samples underwent drying in an oven at 105 °C for 24 h. Following the drying process, the samples were re-weighed using the balance to ascertain the soil moisture content, calculating the difference in weight before and after drying. Herbaceous coverage, abundance and litter content in row 4 were evaluated using herbaceous samples from 0.5 × 0.5 m plots. Shrub characteristics documented included plant name, height, crown width (measured in both east-west and north-south directions) and coverage and abundance within each soil moisture quadrat. Real-time kinematic (RTK) positioning was employed to accurately determine the locations of plants (including latitude, longitude and elevation) and the vertices of each quadrat. Topographic factors, including height difference, slope direction and aspect for each quadrat, were computed using digital elevation models (see Section 2.3.2).

### 2.3. Data Analysis

#### 2.3.1. Soil Moisture Division

Previous research has shown that herbaceous plants in the study area primarily rely on soil moisture within the 0 to 40 cm depth range [33,34]. Furthermore, it has been observed that approximately 80% of the roots of sand-binding shrubs are distributed between 40 and 200 cm, with about 10% of the roots extending between 200 and 300 cm [35,36]. Building upon these findings, this study categorized soil moisture into three layers for analysis: the surface layer (0–40 cm), the middle layer (40–200 cm) and the deep layer (200–300 cm) to investigate its relationship with topographic and vegetation factors.

#### 2.3.2. Calculation of Topographic Factors

**Figure 2:**
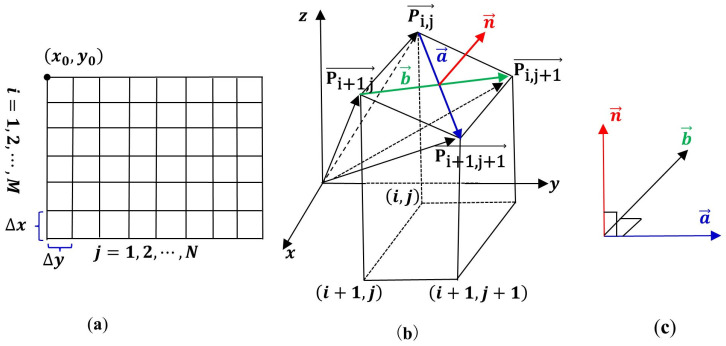
Schematic diagram of grid model space vector analysis. Panel (**a**) shows the mesh model, panel (**b**) illustrates the computational graph of vector $\vec{P_{\mathrm{ij}}}$, while panel (**c**) depicts the fundamental vector $\vec{a}$ and $\vec{b}$.

**Figure 3:**
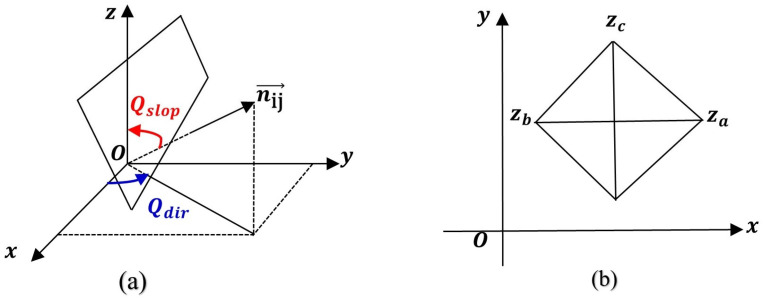
Schematic diagram of slope aspect, slope direction (**a**) and grid point configuration of small quadrat (**b**).

Topographic factors analyzed in this study include height difference, slope direction and aspect. These factors were computed using a digital elevation model, which utilized the latitude, longitude and elevation coordinates of the four vertices of each quadrat [37]. The calculation procedure is outlined as follows (refer to Figure 2 and Figure 3 for visual aids):

The height difference for each quadrat was calculated by averaging the elevations of its four vertices and then subtracting the minimum elevation found within the experimental plot. The slope aspect and direction for each quadrat were calculated based on the principles of spatial analytic geometry. In the mesh model (Figure 2a), ∆x and ∆y denote the unit lengths, with $\left(x_{0},y_{0}\right)\;$representing the origin coordinates of the experimental plot. The vector $\vec{P_{\mathrm{ij}}}$ (Figure 2b) is a standard vector, $\vec{P_{\mathrm{ij}}}$ calculated using Equation (1).

(1) $$\vec{P_{\mathrm{ij}}}=\left(x_{0}+\left(i-1\right)\cdot ∆x,y_{0}+\left(i-1\right)\cdot ∆y,z_{ij}\right)$$

The fundamental vectors $\vec{a}$ and $\vec{b}$ (Figure 2c) are calculated with Equation (2).

(2) $$\left\{\begin{array}{c} \vec{a_{\mathrm{ij}}}=\vec{P_{i+1,j+1}}-\vec{P_{i,j}}=\left(∆x,∆y,z_{i+1,j+1}-z_{i,j}\right)\\ \vec{b_{\mathrm{ij}}}=\vec{P_{i,j+1}}-\vec{P_{i+1,j}}=\left(-∆x,∆y,z_{i,j+1}-z_{i+1,j}\right) \end{array}\right.$$

The unit normal vector $\vec{n_{\mathrm{ij}}}$ is equal to the cross product of vectors $\vec{a}$ and $\vec{b}$, as given in Equation (3).

(3) $$\vec{n_{\mathrm{ij}}}=\vec{a}\times \vec{b}=\left|\begin{array}{ccc} \vec{i} & \vec{j} & \vec{k}\\ ∆x & ∆y & z_{i+1,j+1}-z_{i,j}\\ -∆x & ∆y & z_{i,j+1}-z_{i+1,j} \end{array}\right|\;=\left(\begin{array}{c} ∆y\left(z_{i,j+1}-z_{i+1,j}-z_{i+1,j+1}-z_{i,j}\right),\\ -∆x\left(z_{i,j+1}-z_{i+1,j}-z_{i+1,j+1}-z_{i,j}\right),2∆x∆y \end{array}\right)$$

Were, i=1,2,⋯,M, j=1,2,⋯,N.

The slope aspect $Q_{slop}$ and slope direction $Q_{dir}$ are calculated using the unit normal vector $\vec{n_{\mathrm{ij}}}$. The slope aspect $Q_{slop}$ is equal to the angle between the unit normal vector $\vec{n_{\mathrm{ij}}}$ and z-axis (Figure 3a). If the grid is square, then $Q_{slop}$ is calculated using Equation (4).

(4) $$\left\{\begin{array}{c} U=\frac{\partial_{z}}{\partial_{x}}=\frac{z_{a}-z_{b}}{dis}=\frac{\sqrt{2}\left(z_{a}-z_{b}\right)}{2∆xy}\\ V=\frac{\partial_{z}}{\partial_{y}}=\frac{z_{c}-z_{d}}{dis}=\frac{\sqrt{2}\left(z_{c}-z_{d}\right)}{2∆xy}\\ Q_{slop}=arctan\sqrt{U^{2}+V^{2}} \end{array}\right.$$

where $z_{a}$, $z_{b}$, $z_{c}$ and $z_{d}$ (Figure 3b) represent the height difference at the four grid point of the basic unit, and ∆xy is the basic unit length of grid.

The slope direction $Q_{dir}$ is equal to the angle between the projection of the unit normal vector $\vec{n_{\mathrm{ij}}}$ onto the oxy plane and the x-axis (Figure 3a), with the x-axis direction pointing south. If the grid is square, then the slope direction $Q_{dir}$ for each quadrat is calculated as follows:

When U<0, in the formula $\theta ={tan}^{-1}\left(\frac{V}{U}\right)$, if V=0, then θ=0, and $Q_{dir}$ indicates the direction of E (East). If V<0, then $0<\theta <\frac{\pi }{2}$, and $Q_{dir}$ denotes the direction of EN (East by North). If V>0, then $-\frac{\pi }{2}<\theta <0$ and $Q_{dir}$ denotes the direction of ES (East by South). When U>0, in the formula $\theta ={tan}^{-1}\left(\frac{V}{U}\right)+\pi$, if V=0, then θ=π and $Q_{dir}$ indicates the direction of W (West). If V<0, then $\frac{\pi }{2}<\theta <\pi$, and $Q_{dir}$ denotes the direction of WN (West by North). If V>0, then $\pi <\theta <\frac{3\pi }{2}$ and $Q_{dir}$ denotes the direction of WS (West by South). When U=0, in the formula $\theta ={tan}^{-1}\left(\frac{V}{U}\right)$, if V=0, then θ=0 and $Q_{dir}$ indicates a gentle slope. If V<0, then $\theta =\frac{\pi }{2}$, and $Q_{dir}$ denotes the direction of N (North). If V>0, then $\theta =\frac{3\pi }{2}$, and $Q_{dir}$ denotes the direction of S (South).

#### 2.3.3. Statistical Analysis

In this study, Analysis of Variance (ANOVA) analysis was used to investigate significant differences in soil moisture across three depth layers within four micro-geomorphic units. The *p*-values less than 0.05 were considered statistically significant.

A random forest model, implemented in R software (version 4.3.2), was utilized to quantify the impact of topographic and vegetation factors on soil moisture across surface, middle and deep layers. The model employed observational variables associated with topography, shrubs and herbaceous factors as input features, with various soil moisture depths assigned as output variables (Table 1). The Mean Decrease Accuracy (MDA) importance measure algorithm was applied to evaluate the predictive accuracy of soil moisture considering different topographical and vegetation factors [38]. This algorithm consists of the following steps: First, the random forest model was trained by including all input variables to establish a baseline accuracy. Next, a specific input variable was perturbed by randomly shuffling its values while keeping the other input variables constant, thereby disturbing its relationship with the target variable. The model’s prediction accuracy was then recalculated using the perturbed dataset to assess the effect of the perturbed input variable on accuracy. By comparing this accuracy with the baseline accuracy, the reduction in prediction accuracy due to perturbing the input variable was calculated, representing the importance measure of that variable [39,40]. This process was iterated for each input variable to compute the MDA value of each variable, as illustrated in Formula (5).

(5) $$MDA_{j}=Acc_{baseline}-Acc_{{shuffled}_{j}}$$

Here, $MDA_{j}$ represents the mean decrease accuracy value linked to input variable j, with $Acc_{baseline}$ representing the baseline accuracy and $Acc_{{shuffled}_{j}}$ indicating the accuracy after perturbing input variable j.

The importance measure of MDA reflects the extent of each input variable influences the overall accuracy of the RF model. A higher MDA value for a variable indicates its greater importance in predicting accuracy within the model [40]. Therefore, MDA values serve to identify the most critical variables within the model. MDA helps explain the decision-making process of the random forest model, enhancing our understanding of which input variables play a pivotal role in predicting the target variable.

Structural Equation Modeling (SEM) conducted in AMOS (version 27) was employed to evaluate the relationships and impacts of topographic and vegetation factors on soil moisture [41,42]. SEM is built upon the covariance matrix of all variables, with model parameters estimated by minimizing the difference between the predicted covariance matrix of the model and the actual data covariance matrix. It consists of two primary components: the measurement model and the structural model. The structural model elucidates the causal relationships among latent variables, which are theoretical constructs not directly measurable and are typically represented by multiple observed variables. Path analysis is utilized in the structural model to determine the pathways and magnitudes of influence between latent variables. The measurement model delineates the relationship between latent variables and their observed variables, establishing the observed indicators of each latent variable through factor analysis, as depicted in Formula (6).

(6) $$X=\Lambda_{X}\varphi +\delta$$

where X represents the vector of observed variables, while $\Lambda_{X}$ indicates the factor loading matrix connecting latent variables and observed variables. The vector of latent variables is represented by φ, and measurement errors are denoted by δ.

The parameters of SEM were estimated using Maximum Likelihood Estimation (MLE) [43]. Terrain, shrub, herbaceous and soil moisture factors were treated as latent variables, while their corresponding observed indicators were regarded as observed variables. The complete list of latent variables and observed indicators is available in Table 1.

## 3. Results and Analysis

### 3.1. Soil Moisture Variation Characteristics with Depth

**Figure 4:**
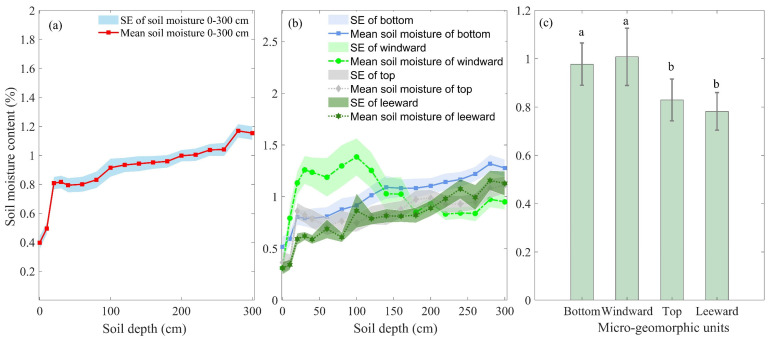
The fluctuation of soil moisture concerning depth within a stable sand dune (**a**), spanning four micro-geomorphic unit categories (**b**), and soil moisture comparison among four micro-geomorphic units (**c**). The continuous lines depict the mean soil moisture content, whereas the dashed lines signify deviations, and shaded regions delineate the standard error in soil moisture across varying depths. In subfigure (**c**), different lowercase letters indicate significant differences.

Figure 4 depicts the variation in soil moisture across the entire fixed sand dune and within the four micro-geomorphic units. Specifically, Figure 4a shows that the average soil moisture content across the fixed sand dune ranges from 0.40% to 1.15%, generally increasing with depth. As shown in Figure 4a, the average soil moisture content at different depths is ranked as follows: deep layer (1.07 ± 0.04) > middle layer (0.89 ± 0.04) > surface layer (0.63 ± 0.03). Furthermore, the interquartile range (IQR) for soil moisture is concentrated within the deep [1.004, 1.153], middle [0.831, 0.951] and surface [0.495, 0.808] layers, indicating variability within each layer. 

As depicted in Figure 4b, soil moisture fluctuation increases with depth at the bottom, top and leeward slope of the dune. Conversely, on the windward slope, moisture content initially increases before decreasing, with the greatest fluctuation observed at a depth of 0–100 cm across all units. Notably, on the windward slope, as depth increases, moisture fluctuation diminishes, underscoring the complex interaction between micro-geomorphic positioning and soil moisture dynamics. ANOVA analysis results indicate significant disparities in soil moisture between the bottom (including the windward slope) and the top (including the leeward slope) of sand dunes (Figure 4c). Among the four types of micro-geomorphic units, the average soil moisture follows a specific order: windward slope (0.98 ± 0.15) > bottom of the sand dune (0.95 ± 0.09) > top of the sand dune (0.81 ± 0.07) > leeward slope of the sand dune (0.75 ± 0.08).

### 3.2. The Distribution Characteristics of Soil Moisture on Four Types of Micro-Geomorphic Units

**Figure 5:**
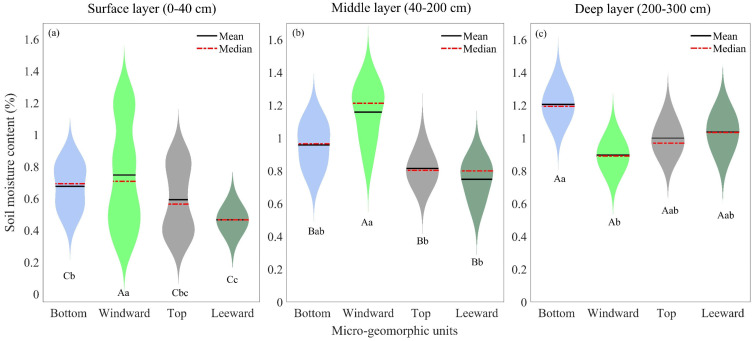
Distribution characteristics of soil moisture across the surface (**a**), middle (**b**) and deep layers (**c**) within four micro-geomorphic unit types. A two-factor ANOVA determined significant differences in soil moisture between these layers, denoted by different uppercase letters. Additionally, a two-factor ANOVA identified significant differences in soil moisture among the four micro-geomorphic unit types, indicated by different lowercase letters.

Figure 5 illustrates the distribution characteristics of soil moisture across three layers within four micro-geomorphic units on a fixed sand dune. Figure 5 demonstrates that soil moisture levels are highest in the deep layer, intermediate in the middle layer and lowest in the surface layer. The soil moisture levels in the middle and deep layers of the four types of micro-geomorphic units exhibit a relatively concentrated pattern, whereas the surface layer soil moisture on the bottom, windward and top of the sand dunes displays a bimodal or multi-peak distribution pattern.

The study utilized Analysis of Variance (ANOVA) to examine the differences in soil moisture levels across the three layers and among the four micro-geomorphic units. Results indicated significant differences in soil moisture among the three layers (F=26.49, df=2, p<0.001), the four types of micro-geomorphic units (F=6.56, df=3, p<0.001) and their interaction (F=2.68, df=6, p<0.05). Specifically, surface layer soil moisture differed significantly (i) between the bottom and windward slope, as well as (ii) between the top and leeward slope (F=6.264, df=3, p<0.001). Middle layer soil moisture showed significant differences between the windward slope and bottom, top and leeward slope (F=3.932, df=3, p<0.05). Similarly, deep layer soil moisture varied significantly between bottom and windward slope, as well as between the top and leeward slope (F=2.74, df=3, p<0.05). Moreover, soil moisture levels varied across the four types of micro-geomorphic units in the three layers. In the surface layer (Figure 5a), the highest soil moisture was observed on the windward slope (0.87 ± 0.18), followed by the bottom of sand dunes (0.68 ± 0.08), the top of sand dunes (0.61 ± 0.09) and the leeward slope (0.47 ± 0.06). In the middle layer (Figure 5b), the highest soil moisture was observed on the windward slope (1.15 ± 0.20), followed by the bottom of sand dunes (0.96 ± 0.10), the top of sand dunes (0.81 ± 0.06) and the leeward slope (0.75 ± 0.09). The deep layer (Figure 5c) had the highest soil moisture at the bottom of sand dunes (1.20 ± 0.08), followed by the leeward slope (1.04 ± 0.10), the top of sand dunes (1.01 ± 0.07) and the windward slope (0.89 ± 0.08).

### 3.3. The Important Measure of Topographic-Vegetation Factors on Soil Moisture at Three Layers Using a Random Forest Model

Table 2 presents the ranking of the influence of topographic and vegetation factors on soil moisture in three layers, based on the Mean Decrease Accuracy (MDA) importance measures derived from a Random Forest Model (RF). Table 2 identifies the six variables with the highest MDA importance degrees, illustrating the predominant impact of topographic factors on soil moisture in all layers. Particularly, the height difference has a significant effect on soil moisture in the surface and middle layers, with importance degrees of 46.01% and 36.40%, respectively. Slope direction is the predominant factor affecting deep layer soil moisture, with an importance value of 48.41%. Additionally, factors related to shrubs and herbaceous plants, specifically abundance, coverage and biomass, also play significant roles in the surface and middle layers, with importance values exceeding 20%. Notably, in the deep layer, topographic factors—specifically slope direction, height difference and slope aspect—are crucial, each with importance values exceeding 35%. Furthermore, in the deep layer, significant contributions to soil moisture come from shrub coverage, abundance and biomass, with importance values of 32.27%, 26.08% and 30.13%, respectively.

### 3.4. The Direct and Indirect Effects of Topographic-Vegetation Factors Affect Soil Moisture 

This study employed Structural Equation Modeling (SEM) to explore the direct and indirect relationships between topographic and vegetation factors and soil moisture in fixed dunes. Based on the findings presented in Section 3.3, it was posited that topographic factors exert both direct and indirect effects on soil moisture. Directly, they can impact the variation of soil moisture, while indirectly, they can influence soil moisture through interaction with shrub and herbaceous factors. Furthermore, this study hypothesized that both shrub and herbaceous factors not only interact with each other but also both contribute to variations in soil moisture. To test these hypotheses, the SEM model included explanatory variables such as slope direction, slope aspect and the height difference within each small quadrat. Vegetation factors were divided into shrub-related factors (including shrub coverage and abundance) and herbaceous factors (encompassing herbaceous coverage, abundance, biomass and total litterfall).

**Figure 6:**
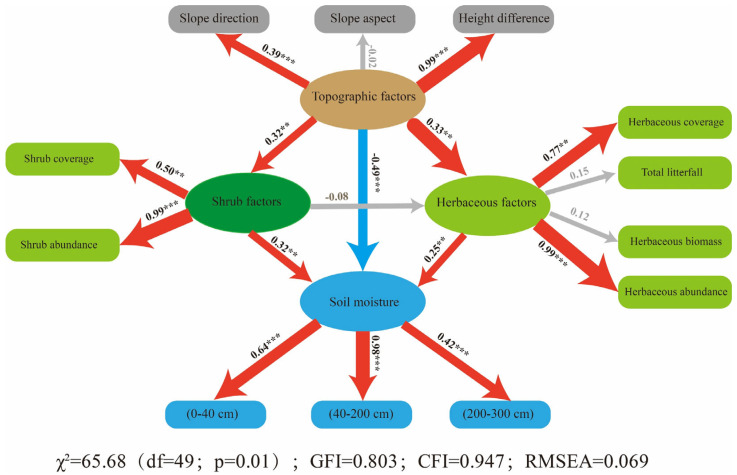
Structural Equation Modeling (SEM) illustrating the effects of topographic, shrub and herbaceous factors on soil moisture in fixed sand dunes of the Tengger Desert, China. Red arrows indicate statistically significant positive correlations, blue arrows indicate statistically significant negative correlations and grey arrows represent non-significant relationships. The numbers adjacent to the arrows show the standardized path coefficients, and the width of the arrows reflects the strength of these relationships. Statistical significance levels are denoted by ‘**’ for *p* < 0.05 and ‘***’ for *p* < 0.01, respectively.

The findings of the study demonstrated that the SEM models (Figure 6) were statistically significant, as confirmed with the $\chi^{2}$ test (p<0.01). Topographic factors were found to have a significantly negative effect on soil moisture, evidenced by a path coefficient of −0.49 (p<0.01). In contrast, shrub and herbaceous factors exhibited positive influences on soil moisture, with path coefficients of 0.32 (p<0.05) and 0.25 (p<0.05), respectively. Furthermore, the analysis indicated that topographic factors had a larger impact on soil moisture than vegetation factors. Additionally, topographic factors had a significant positive effect on both shrub and herbaceous factors, with path coefficients of 0.32 (p<0.05) and 0.33 (p<0.05), respectively. Specifically, among topographic factors, height difference and slope direction exhibited the most pronounced effects, with path coefficients of 0.99 (p<0.01) and 0.39 (p<0.01), respectively. For shrub variables, abundance and coverage were the most influential, with path coefficients of 0.99 (p<0.01) and 0.50 (p<0.05), respectively. Herbaceous abundance and coverage were identified as key factors, with path coefficients of 0.99 (p<0.001) and 0.77 (p<0.05), respectively. In summary, the insights provided by the SEM model regarding the significant effects of topographic and vegetation factors on soil moisture align with the findings from the random forest analysis presented in Section 3.3.

## 4. Discussions

### 4.1. Variability in Soil Moisture with Three Depth Layers across Four Types of Micro-Geomorphic Units

The research provides a detailed analysis of soil moisture variability across three depth layers and four micro-geomorphic units within a fixed sand dune located at the southeastern margin of the Tengger Desert, China. The findings reveal an increase in soil moisture within 0–300 cm depth, which is consistent with the anticipated phenomenon that deeper layers retain more moisture due to lower evaporation and infiltration rates [3,44,45]. The vertical moisture gradient from the surface to deeper layers is likely influenced by factors including the distribution of vegetation, soil texture, porosity and evaporation rates. The quantified average moisture contents demonstrate significant stratification in moisture distribution across the dune, which may affect root penetration, microbial activity and nutrient cycling. The observed bimodal or multi-peak distribution pattern of soil moisture in the surface layer, particularly at the dune’s bottom, windward slope and top, reflects variability in moisture inputs and losses. Such patterns could be shaped by soil properties such as particle size and organic matter content, which influence the soil’s water-holding capacity [46,47]. Significant variations in soil moisture content between the surface, middle and deep layers, as determined by ANOVA analysis, suggest that these layers possess distinct hydrological characteristics [48,49,50]. Furthermore, the variability observed within these layers, as indicated by the interquartile range, provides insights into the heterogeneity of soil moisture, likely due to local differences in soil composition, texture, organic matter content and distribution of vegetation [51,52,53].

This study demonstrated that soil moisture levels within sand dunes exhibit spatial variability, influenced by their location on the dune. Soil moisture in the surface and middle layers is found to be highest on the windward slope, whereas the deepest layer’s soil moisture reaches its maximum at the bottom of the dunes. These findings suggest that factors such as wind direction and micro-geomorphic characteristics significantly influence the spatial distribution of soil moisture [54,55]. Soil moisture content displays a gradient, decreasing from the windward to the leeward slope, which highlights the influence of the dune’s morphology and external factors such as wind and exposure to sunlight. The windward slope exhibits heightened moisture levels, presumably influenced by wind-driven precipitation, in contrast to the leeward slope, which experiences reduced moisture input and increased evaporation rates [56,57,58]. The elevated moisture content at the bottom of the dune may result from the accumulation of water runoff and the protective influence of dense sand-binding vegetation, which mitigates evaporation in this sheltered zone. The distinct moisture distribution pattern on the windward slope, characterized by an initial increase followed by a decrease with depth, suggests a complex interplay of factors including wind erosion, sand movement, sand deposition and hydrodynamics [59,60]. Moreover, the noted decline in moisture variability with depth, notably pronounced on the windward slope, indicates a stabilizing effect at greater depths crucial for sustaining the stability of sand-binding vegetation and the overall structural robustness of the dune. The analysis of soil moisture across four types of micro-geomorphic units has illuminated the significant impact of landscape features on moisture dynamics. These patterns of soil moisture distribution are critical to understanding both ecosystem functioning and effective management strategies in arid environments [61,62,63]. Importantly, our findings reveal the complex and variable nature of soil moisture distribution within sand dune ecosystems. This complexity underscores the need for nuanced approaches in the restoration and management of sand-binding vegetation.

### 4.2. The Important Measures of Topographic-Vegetation Factors on Soil Moisture across Different Layers

This study underscores the pivotal role of topographic factors, such as height differences and slope direction, in influencing soil moisture variability. These findings reveal the intricate relationship between topography and soil moisture, demonstrating that soil moisture distribution results not only from meteorological conditions but also from the landscape’s physical characteristics [3,47,64]. In particular, the significant impact of height differences on surface and subsurface soil moisture primarily arises from gravitational water movement and subsequent moisture accumulation at lower elevations, a crucial mechanism for enhancing water retention in soil profiles [65,66]. Similarly, the investigation into slope direction reveals its substantial influence on the moisture content of deeper soil layers, primarily through the modulation of solar radiation exposure and evaporation rates, which vary according to the orientation of the slope [67,68]. Plant roots exhibit varying distribution across different slopes, slope directions and height differences. Preferential flow also takes place around the roots, thereby augmenting the moisture content in the deep soil layers through this mechanism [12,13]. These insights enhance our understanding of the dynamic interactions between topography and soil moisture regimes, offering valuable implications for the management and conservation of micro-geomorphic unit ecosystems.

Our findings demonstrate that specific vegetation factors, notably abundance, coverage and biomass of shrubs and herbaceous, significantly influence soil moisture content at various depths. This notable impact stems from the dual function of vegetation: firstly, its structural attributes, such as leaf density and canopy cover, effectively intercept rainfall and mitigate evaporation through shading; secondly, root systems and organic matter contribute to soil structural improvements, thereby enhancing moisture retention capabilities [2,4,46,69]. Furthermore, our analysis elucidates the pivotal influence of shrub biomass on the moisture content of deeper soil layers, underscoring the root systems’ integral function in facilitating water penetration and bolstering soil’s water-holding capacity. These observations are congruent with prior studies that delineate the complex interplay between soil moisture dynamics and the multifaceted influences of both physical and biological determinants [70].

Comprehending the paramount influence of topography and vegetation on soil moisture dynamics is critical for multiple reasons [2,47]. First, this understanding provides critical insights into water availability within desert ecosystems, which is pivotal for supporting plant growth, preserving biodiversity and maintaining habitat quality. Second, these insights have profound implications for water management practices [71,72,73], especially critical in the context of climate change, which necessitates adaptive water conservation and management strategies to address altering precipitation patterns. Furthermore, this study highlights the complex and significant effects of topography and vegetation on soil moisture at various depths. By providing a detailed ranking of these influences, the study contributes important insights into the mechanisms driving soil moisture variability, essential for windproof sand fixation and ecological reconstruction of sandy areas, ensuring sustainable resource utilization while minimizing degradation of sand-binding vegetation in desert ecosystems [31,55].

### 4.3. The Direct and Indirect Effects of Topographic-Vegetation Factors on Soil Moisture

The use of Structural Equation Modeling (SEM) in this study clarifies the intricate relationships between topographic and vegetation factors and their impacts on soil moisture in fixed dunes, offering a detailed understanding of these interactions [74]. The SEM analysis, validated by a significant $\chi^{2}$ test, offers a robust framework for interpreting the direct and indirect effects of these variables on soil moisture dynamics. The identification of a significant negative direct effect of topographic factors on soil moisture, indicated by a path coefficient of −0.49, suggests that topographic variations may lead to increased drainage or evaporation, consequently reducing soil moisture [3,75,76]. The positive indirect effect of topographic factors on vegetation factors, which subsequently positively influence soil moisture, underscores the complex interplay in which topography may facilitate vegetation types that are more effective in retaining soil moisture [3,4,46,77]. The SEM analysis supports the initial hypotheses, demonstrating that topographic factors exert both a direct negative impact on soil moisture and an indirect positive effect through their influence on vegetation factors [3,4]. This dual role underscores the complexity of interactions in dune ecosystems. The negative direct path implies that specific topographic configurations may result in decreased soil moisture, possibly due to elevated runoff or evaporation rates. Conversely, the positive indirect effect via vegetation suggests that topography can foster favorable conditions for vegetation growth, which, in turn, enhances soil moisture through shading and reduced evaporation.

This study reveals the notable positive impact of shrub and herbaceous factors on soil moisture, as evidenced by path coefficients of 0.32 and 0.25, respectively. The findings underscore the critical role of shrubs and herbaceous in augmenting soil moisture, facilitated by mechanisms such as transpiration pull, shading and the contribution of organic matter to soil structure [3,4,78]. The observed influence of both the coverage and abundance of shrub and herbaceous vegetation suggests that areas with higher vegetation density are likely to retain more soil moisture by reducing surface runoff, enhancing infiltration and minimizing soil evaporation through shading [79,80,81,82]. These findings align with the plant ecological niche theory [83], which emphasizes the critical role of vegetation in regulating the water cycle and facilitating interactions between soil, plants and the atmosphere. Furthermore, the substantial positive effects of topographic factors on the coverage of both shrub and herbaceous vegetation, indicated by path coefficients of 0.32 and 0.33, respectively, suggest that topography indirectly influences soil moisture by dictating the distribution and abundance of vegetation [11,84,85]. This underscores the imperative to account for topographic variations during the reconstruction and restoration of sand-banding vegetation, particularly in desert ecosystems where height difference, slope direction and aspect significantly influence microclimates and vegetation dynamics.

In conclusion, elucidating the direct and indirect effects of topographic and vegetation factors on soil moisture dynamics is pivotal for the sustainable management and conservation of arid and semi-arid desert ecosystems. Such insights are instrumental for the strategic planning of ecological restoration endeavors. They facilitate the selection of vegetation types conducive to enhancing soil moisture retention and promoting dune stabilization, taking into account the nuanced variations in micro-topography, such as height differences, slope direction and aspect. Moreover, this comprehensive understanding is critical for forecasting the potential impacts of climate change on these fragile ecosystems [86,87], with a particular emphasis on the ramifications of altered precipitation patterns and escalating temperatures.

## 5. Conclusions

This study employed random forest analysis and Structural Equation Modeling (SEM) to identify key factors influencing soil moisture and to elucidate the direct and indirect interactions between topography, vegetation and soil moisture within the Tengger Desert’s fixed sand dunes, in China. The investigated soil profiles showed evident heterogeneity in soil moisture, with significant variations observed across surface, middle and deep layers, as well as between windward and leeward slopes and at the tops and bottoms of sand dunes. Notably, moisture content exhibits a progressive increase from the surface down to a depth of 300 cm. Concurrently, this gradient is characterized by a diminishing variability in moisture levels, which stabilize in the deeper layers. Soil moisture content is highest in the surface and middle layers on the windward slope, whereas it peaks in the deep layer at the bottom of the sand dunes. A distinctive pattern is evident on the windward slope, where soil moisture initially increases and then decreases. Both topographic and vegetation factors significantly impact soil moisture across the three depth layers. Specifically, topographic variables such as slope direction and height differences exert a considerable impact on soil moisture. Similarly, vegetation attributes, mainly including the coverage and abundance of shrubs and herbs, markedly affect soil moisture levels. Topographic factors demonstrate significant negative direct effects on soil moisture, while also exerting positive indirect effects mediated through vegetation. Notably, the coverage and abundance of shrubs and herbaceous plants directly enhance soil moisture.

These findings offer valuable insights for ecological management and the formulation of effective strategies to conserve soil moisture in the study area. Future endeavors should systematically and specifically tailor approaches to the diverse micro-geomorphic units of sand dunes and soil moisture characteristics for establishing vegetation to stabilize the sand. While our study elucidates the pathways through which topographic and vegetative factors influence soil moisture in the Tengger Desert of China and their primary determinants, the generalizability of these effects to other arid desert ecosystems remains uncertain. Moreover, variations in soil particle size (particularly the finer fractions), organic matter content, plot size, climatic conditions and groundwater levels may also influence the accuracy of research conclusions. Subsequent investigations should further integrate factors such as soil texture, groundwater levels, climate, long-term monitoring and land use changes across different regions to explore the relationship between soil moisture, topography and vegetation in arid ecosystems, thus elucidating their universality, disparities and underlying mechanisms.

## Figures and Tables

**Table 1:** Variables in random forests and structural equation models.

Input/Output	Category	Name	Units
Input variables	Topographic factor	Slope direction	rad
		Slope aspect	rad
		Height difference	m
	Shrub factors	Shrub coverage	%
		Shrub abundance	%
	Herbaceous factors	Herbaceous coverage	%
		Herbaceous abundance	%
		Total litterfall	g
		Herbaceous biomass	g
Output variables	Soil moisture	Surface (0–40 cm)	%
		Middle (40–200 cm)	%
		Deep (200–300 cm)	%

**Table 2:** Importance measures based on Mean Decrease Accuracy (MDA) from a Random Forest Model (RF): ranking the effects of topographic and vegetation factors on soil moisture across three layers.

Ranking	Surface Layer (0–40 cm)	Middle Layer (40–200 cm)	Deep Layer (200–300 cm)
1	Height difference (46.01%)	Height difference (36.40%)	Slope direction (48.41%)
2	Shrub abundance (45.33%)	Shrub abundance (32.07%)	Height difference (42.62%)
3	Shrub coverage (36.30%)	Herbaceous coverage (25.90%)	Slope aspect (34.74%)
4	Biomass (31.25%)	Shrub coverage (24.23%)	Shrub coverage (32.27%)
5	Herbaceous coverage (29.65%)	Total litterfall (23.84%)	Herbaceous biomass (30.13%)
6	Slope aspect (24.85%)	Herbaceous biomass (22.22%)	Shrub abundance (26.08%)

## Data Availability

The original contributions presented in the study are included in the article’s data. Further inquiries can be directed to the corresponding author and will be available upon reasonable request.
